# Determining the tactical and technical level of competitive tennis players using a competency model: a systematic review

**DOI:** 10.3389/fspor.2024.1406846

**Published:** 2024-07-17

**Authors:** Miguel Crespo, Rafael Martínez-Gallego, Ales Filipcic

**Affiliations:** ^1^ Development Department, International Tennis Federation, London, United Kingdom; ^2^ Department of Sport and Physical Education, University of Valencia, Valencia, Spain; ^3^ Faculty of Sport, University of Ljubljana, Ljubljana, Slovenia

**Keywords:** tennis, skills, knowledge, game situations, match performance, analysis

## Abstract

**Introduction:**

The aim of this systematic review is to provide an evidence-based synthesis of the literature on the topic of technical and tactical competencies of tennis players and to answer the following research questions: (1) What is the state of the art of research on technical and tactical competencies (i.e., skills and knowledge) and tennis; (2) What are the most important topics related to technical and tactical competencies in tennis players.

**Methods:**

Electronic searches were conducted in Web of Science, PubMED and SPORTDiscus (August to September 2023). This systematic review was conducted in accordance with PRISMA guidelines. To reduce risk, all published literature was searched and primary studies were included. The search terms included skills or competencies, match or play, player and tennis and excluded studies on non-competitive tennis players—notation analysis, AI method, systematic review and validation of tools.

**Results and discussion:**

Of the 390 publications found in these searches, 13 articles were considered relevant and included in this study. They were divided into three categories: (1) technical-tactical skills, (2) match situations and (3) match performance. There was clear evidence that there is a test instrument for analyzing tactical-technical skills that has sufficient reliability and validity and is of practical value to tennis coaches. The development of tactical-technical skills is influenced by method (variability between/within skills), conditions (court size, ball type) and areas of development (situational awareness, anticipation, decision making). There are differences in match and stroke performance between different quality groups (professionals, juniors), which can also be influenced by mental strength. For a comprehensive study of tennis players’ abilities, the use of modern technologies is possible and necessary in the future. Future research should focus on the creation of competency models for the playing level of tennis players, which could include at least three key elements: (1) key competencies, (2) description of standards, (3) evidence.

## Introduction

Tennis is a complex, dynamic and open sport where players strive to achieve a certain level of play by effectively implementing the different components of the game such as the strategy, condition, mentality, and technique to increase the probability of improving their performance and eventually winning more matches (1, 2).

In this context, the players’ level of play and their success in tennis, as in many other aspects of life and sport have a considerable importance and they are very closely interrelated. The level of play of a given tennis player can be understood as a multifaceted construct, encompassing a range of physical, mental, and technical skills. It also includes factors related to the strategical and tactical knowledge of the player, as well as external factors such as equipment and coaching (3). For instance, from a psychological perspective research has shown that tennis players believe that ability, maintaining a positive attitude, being task oriented, and focusing on effort were the primary causes of the improvement of their level of play and thus their success (4). As indicated by Elliott (5) “*Success in tennis requires a mix of player talent, good coaching, appropriate equipment, and an understanding of those aspects of sport science pertinent to the game*” (p. 392). From a biomechanics perspective, this author considers that “*Success in tennis is greatly affected by the technique a player uses and biomechanics plays an integral role in stroke production*” (p. 392).

As per the tactical components of the game, the level of the players and their success has also been a relevant topic of research. Authors have investigated level of play and success factors in the tactical space for junior tennis players (6). In this context, the purpose and nature of adequate competition has been defined, discussed and analysed as a fundamental aspect to ensure the proper pathway to improve the level of play and facilitate success from 10-and-under tennis onwards (7). From a conditioning and movement perspective, the nuances of positioning, displacement, recovery according to the physical and physiological demands of the game have also attracted the interest of researchers (8).

When adopting a developmental approach on the factors that affect the players’ level and success in tennis, researchers have also explored the importance of performances in youth and junior competition as an indicator of later success in tennis (9). Furthermore, this topic, the level of play and the success in tennis has not only been explored individually from the players perspective but, also, when considering a wider view as a performance indicator for nations when researchers have analysed the factors which explain nations’ success in tennis (10). In this context, the tournament structure and geography of tennis events and how these affect the level of play and the subsequent success of players has also been a subject of investigation (11).

From a long-term player pathway approach, at the grassroots level of play, tennis success has been considered on a developmental perspective (12). The level of play and success of young players stepping onto the court for the first time may be considered in terms of skill acquisition, enjoyment, and the development of a passion for the sport (13). According to the various tennis-specific player development models (14, 15), early success is linked to the attainment of a certain level of play defined by the acquisition of fundamental movement skills and the cultivation of a positive attitude towards physical activity. This foundational achievement of a given level of play and the subsequent success set the stage for a lifelong engagement with tennis, irrespective of future competitive pursuits (16).

In addition to tactical and technical skills, a tennis player's level of play (17) and success has been shown to be influenced by many internal factors, such as the level of psychological (18) and conditioning preparation (19), level of experiences, daily performance, and external factors, which primarily include the opponent (20), playing surface (21), tournament round, weather conditions (22), referee decisions, and spectators (23).

Competency and competency model are common terms that have received considerable attention from researchers in different fields. The competency model is a framework widely used in the accreditation of professions as organisations have shown interest in the development of competencies for different occupations (24). For instance, in the sports science and medicine context different organisations have created a group of prescribed competencies or indicators related to how the professions of these sports professionals are defined and assessed. The competency model is increasingly being used in sport to determine success and efficiency (25). Specifically in the case of the sport domain, Siedentop, Hastie and van der Mars (26) defined a competent athlete as someone who has developed sufficient skills to participate satisfactorily in games and activities, who understands and can execute strategies appropriate to the complexity of the activity, and who is a knowledgeable athlete with specific cognitive, psychomotor, tactical and technical skills. In racket sports, competence is defined as the knowledge, skills, behaviours, attitudes and abilities to do something successfully or efficiently, including technical skills (e.g., shot accuracy), tactical awareness (e.g., decision making), physical fitness (e.g., agility) and mental aspects (e.g., concentration) (27). Specifically, skills in tennis have been understood as specific actions (e.g., shots and movement patterns) that can be developed through training and practice (28).

The use of competencies and competency models for the identification and categorisation of tennis players has been a usual policy by many tennis organisations as part of their player development strategy. For instance, as an example, in the case of the United States Tennis Association (USTA), the American Development Model (ADM) is a competency-based framework aimed at providing guidelines to work with players at the different levels of the game (29). Furthermore, the International Tennis Federation (ITF) as well as a considerable number of national associations are also using a competency-based structure in their coach education programmes to facilitate the coaches’ pathway (30). Being able to define and allocate the appropriate player competencies to the different levels of the game is of paramount importance to enhance the quality of both coaching and coach education programmes (31).

Research on specific competencies in the tennis domain is somehow fragmented and unclear as it covers a multiplicity of subtopics and approaches that may generate confusion in this relevant issue. Some studies have explored the professional competencies of tennis coaches (32, 33). As per the players, evidence in tennis has focused on the measurable and observable results that demonstrate a player's competencies. These competencies have been understood as the combination of their knowledge and skills as related to the game in general and to match play in particular. It involves performance indicators (e.g., percentage of strokes or points and success, position, speed and placement, position and movement of the player,…) and other data (temporal characteristics, heart rate, heart rate variability,…) that provide objective information about a player's efficiency and effectiveness (34).

Evidence can be collected through various methods. In the past, researchers analysed tennis play using time-consuming video analysis. Today, various tools are available for in-depth analysis of tennis play, including technologies that automatically track the ball and the tennis player (Hawk Eye, FoxTen) or wearable devices that are attached to the tennis racket or the player (35). Researchers have analysed different areas of the game and gathered increasingly detailed information, such as the relationship between the players’ competitive level and their ranking (36), the playing characteristics on different surfaces (37), a better understanding of the differences between players of different levels of play (6, 38, 39), and between winning and losing players (40, 41), or between players of both genders (38, 42).

Some of the studies have focused on the search for specific performance indicators that influence tennis performance (43, 44), methods to determine stroke type and external load (Whiteside et al.), hitting load and running distance, and movement velocity (45). Research has also analysed serve position, velocity, projection angle, landing position, and relative position of the player when executing a serve (46), as well as movement characteristics, groundstroke velocity, and net clearance of strokes (44).

In terms of studies related to level of play, tennis, a sport known for its elegance and competitive spirit, unfolds on a vast spectrum of levels, ranging from grassroots participation to the pinnacle of professional tournaments (47). Researchers have tried to delve into the diverse competences needed to achieve success at various levels of tennis play, acknowledging the subjective nature of accomplishment, the evolving criteria that define it, and aiming at exploring objective features that would allow to better determine the various levels of the game (48).

Moving up the ladder to junior and amateur levels, success begins to intertwine with competitive achievement. Here, players may gauge success by their ability to navigate local and regional tournaments, climb the rankings, and secure scholarships to advance their education while pursuing their tennis aspirations (49). As an example, the work of Gould et al. (50) among others, sheds light on the importance of appropriate player-parent relationships and the role of positive coaching in shaping success at these levels. Success is not only about winning matches but also about overcoming challenges, refining skills, and establishing a competitive identity.

Authors such as Pummell & Lavallee (51) have explored the path of players in the junior-to-senior transition as moving into the professional realm, success undergoes a paradigm shift, with a heightened emphasis on rankings, Grand Slam victories, and financial gains. Scholars such as Li et al. (52) emphasize the significance of mental resilience, adaptability, and strategic career planning in achieving success at the professional level. Success in professional tennis is intricately linked to a player's ability to navigate the complex interplay of physical fitness, mental toughness, and strategic decision-making in an intensely competitive global landscape.

An interesting area of research has been the determination of the different levels of what can be considered quality in tennis. In most cases, the individual level of tennis players has been determined using rankings (53–55), player experience (56), and analysis of selected performance indicators (57, 58).

From a content perspective, it seems obvious that there is a considerable body of research on aspects related to the technical (biomechanical) and tactical (strategical) components of the game. The studies focused on stroke production (technique) and match performance (tactics) have received a considerable attention from researchers when investigating the main performance indicators that determine the various levels of play.

Despite the importance of the definition and the categorisation of the specific competencies that players need to master at the different levels of play, it can be observed that the state of research provides a fragmented view on this topic with a considerable relevance of studies on skills and performance indicators but a lack of research and application on competency-based analysis and understanding of the various levels of play that define a player.

Thus, the aim of this systematic review is to provide an evidence-based synthesis of the literature on the topic of technical and tactical competencies of tennis players and answer the following research questions: (1) Which is the state of research on technical and tactical competencies (i.e., skills and knowledge) in tennis; (2) Which are the main themes related to technical and tactical competencies as applied to tennis players.

## Methods

### Search strategy

This systematic review was conducted in accordance with PRISMA guidelines (59). An extensive search of the research literature was conducted to systematically review the competencies of tennis players. the search spanned from august to september 2023 and included literature from online databases such as web of science, pubmed, and sportdiscus. The search terms used in the electronic database were (“skills” or “competencies”) and (“match” or “play”) and “player” and “tennis” (Table 1). In addition, the reference lists of included articles were manually reviewed to identify additional relevant studies for review.

**Table 1:** Databases, search items and inclusion/exclusion criteria overview.

Databases	Search terms	PICOS	Inclusion criteria	Exclusion criteria
Web of science Pub Med SportDiscus	Tennis, competencies, skills, match, game, player	Population	Male and female tennis players at junior, national, or international match level	Recreational tennis players
		Intervention	Analysis, validation and evaluation of tactical and technical performance indicators Time-motion characteristics	Physiological and physical demands Psychological and mental predictors
		Comparison	Between tactical-technical performance indicators and effectiveness	Only between one aspect of the game and the success of the players
		Outcome	Useful tool to monitor technical and tactical content; identify differences between junior and professional games	Outcome does not allow generalization at the skill level
** **		Study design	Comprehensive study with multivariate variables	** **

### Inclusion and exclusion criteria

In accordance with the PICOS (Population, Intervention, Comparison, Outcomes, and Study Design) method (60), the following inclusion criteria were used to select articles for this review (Table 1). (1) Inclusion criteria: Male and female tennis players at junior, national or international competition level, (2) Intervention: analysis, validation and evaluation of tactical and technical performance indicators and time characteristics. (3) Comparison: between tactical-technical performance indicators and effectiveness. (4) Results: Useful tool for monitoring technical and tactical content; identification of differences between junior and professional games. (5) Study design: Comprehensive study with multivariate variables.

**Figure 1:**
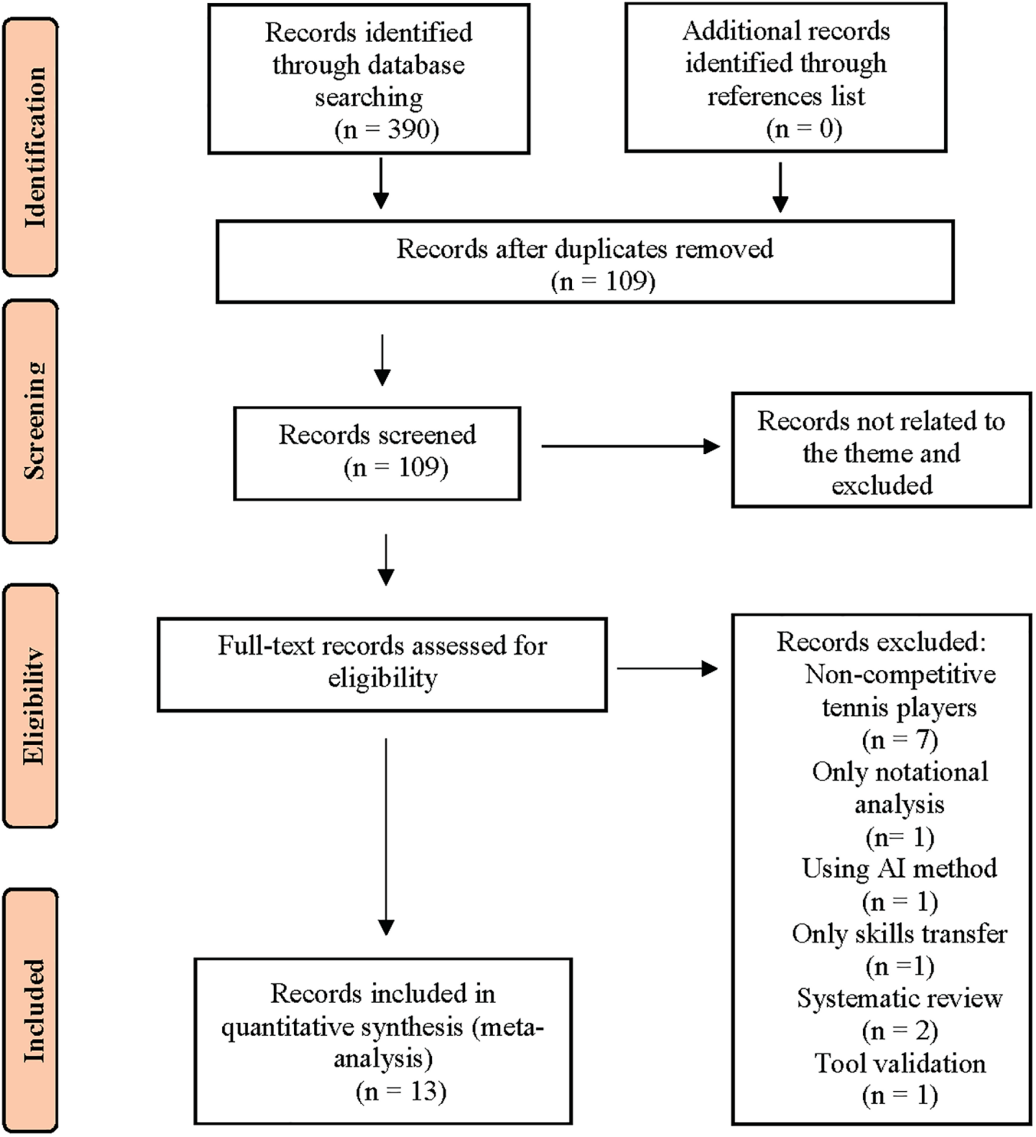
Stages adopted in the systematic selection of articles analysing tennis players competencies and/or skills in match and/or play conditions.

Studies were excluded according to the following criteria: (1) Non-competitive tennis players (understood as recreational players); (2) Physiological and physical demands and psychological and mental predictors; (3) Only between one aspect of the game and the success of the players; (4) Outcome does not allow generalization at the skill level; (5) Studies written in languages other than English and Spanish. A reviewer checked every data set and every document found. The steps applied in the systematic search led to the identification of 13 relevant articles for further analysis, the data of which were obtained or confirmed by the reviewers (Figure 1).

The quality of the applied methodology in the included articles was assessed using the Critical Review Form—Qualitative Studies (61). This tool can be used to evaluate many types of qualitative studies. This method was applied to assess each article according to the following categories: study purpose, literature background, study design, sample, outcomes, data analysis methods, results, conclusions and implications for future research. These questions were assigned a score of either 1 (meet the criteria) or 0 (do not meet the criteria). The seventh and eighth questions were exceptional, as a NR (not registered) score could also be assigned to articles.

An NR score means that no information was available on the reliability or validity of the instruments used in this systematic review. For the fifth question, articles that reported on studies with a sample size of at least 21 were assigned a score of 1, as this was the number required to achieve a statistical power of 0.80 or more to detect a large (one-sided) difference at a 5% significance level. The scores obtained for the 14 questions were totaled for each item, with the NR score counted as 0. Two experts independently assessed the quality of the selected items. Table 2 shows the methodological quality of the studies examined.

**Table 2:** Methodological quality of reviewed articles.

	Question number														
	1	2	3	4	5	6	7	8	9	10	11	12	13	14	Total
Author (year)															
Buszard et al. (62)	1	1	1	1	0	1	NR	NR	1	1	0	1	0	0	8
Caserta and Singer (63)	1	1	1	1	1	NR	NR	NR	1	1	0	1	1	0	9
Chang and Qiu (64)	1	1	1	1	0	NR	1	1	1	1	1	1	0	1	11
Cowden (65)	1	1	1	1	1	1	NR	NR	0	1	1	1	1	1	11
Dimic et al. (66)	1	1	1	1	0	0	NR	NR	1	1	1	1	0	1	9
Féry and Crognier (67)	1	1	1	1	0	0	NR	NR	1	1	0	1	0	0	7
Fitzpatrick et al. (68)	0	1	1	1	1	1	NR	NR	1	1	0	1	1	1	10
Garcia-Gonzalez et al. (69)	1	1	1	1	0	1	NR	NR	1	1	0	0	0	0	7
García-González et al. (70)	1	1	1	1	0	1	1	NR	1	1	1	1	1	0	11
Gimenez-Egido et al. (71)	1	1	1	1	0	1	1	NR	1	1	1	0	1	1	11
Kolman et al. (72)	1	1	1	1	1	1	1	1	1	1	0	1	1	0	12
Kovalchik and Reid (73)	1	1	1	1	1	NR	NR	NR	1	1	1	1	1	0	10
Raschke and Lames (74)	1	1	1	1	1	1	NR	NR	1	1	0	1	1	0	10

NR, not registered; 1 = meet criteria; 0 = does not meet criteria; Questions = (1) Was the aim of the study stated clearly? (2) Was relevant background literature reviewed? (3) Was the design appropriate for the research question? (4) Was the sample described in detail? (5) Was sample size justified? (6) Was informed consent obtained? (7) Were the outcome measures reliable? (8) Were the outcome measures valid? (9) Were results reported in terms of statistical significance? (10) Were the analysis methods appropriate for the research design? (11) Was practical importance reported? (12) Were conclusions appropriate given the study findings? (13) Are there any implications for future research given the results of the study? (14) Were limitations of the study acknowledged and described by the authors?

## Results

In order to facilitate the presentation of the results, this section presents the findings of our study by organizing them according to the two research questions mentioned in the introduction, which refer to the state of research on technical and tactical competencies (i.e., skills and knowledge) in tennis and to the main issues related to technical and tactical competencies in relation to tennis players.

(a) State of research on technical and tactical competencies (i.e., skills and knowledge) in tennis

Table 3 shows the authors of the studies, the number, gender, age, performance level, and tennis experience of the subjects, the research area, and the results reported in the 13 articles included in the review.

(b) Main themes focused on technical and tactical competencies as applied to tennis players.

**Table 3:** Characteristics of the 13 studies reviewed technical-tactical skills, match situations and performance.

Author	Sample size, sex	Age (years) ± SD	Level of performance, playing experience (years), number of participants	Measure(s) of skill(s)	Results
Buszard et al. (62)	Study 1: 7 male, 2 female Study 2: 8 male, 8 female	Study 1: 17.1 ± 3.0 Study 2: male 12.1 ± 0.4; female 12.1 ± 0.9	Study 1: N (*n* = 9) Study 2: N (*n* = 16)	Study 1: Video clips were used to observe 1) the type of tennis shot type, 2) the side of the serve, and 3) the placement of the serve, and to detail the variability between skills and within skills during training. Study 2: Observing the effect of contextual interference (CI) on serve learning in “blocked” (low CI) and “serial” (moderate CI) practice conditions.	Study 1: 6 of 9 players = no differences between-skill variability in serve Within-skill variability: placement > side Accuracy: correlations: between-skill variability (*r* = 0.15, *p* = 0.26, within-skill variability (*r* = 0.16, *p* = 0.24). Study 2: % of hits to target: moderate CI > low CI % serves in: no differences Serve displacement to T: low CI pre-test < post-test Speed: no differences
Caserta and Singer (63)	59 male & female	21.75 ± 4.96		To determine the effects of situation awareness (SA), anticipation (ANT) and decision making (DM) on the perceptual abilities of players in experimental (EG) and control (CG) groups.	SA, A, DM practice improve performance Response speed: E < C Accuracy DM score: E < C No differences in implicit and explicit learning strategies
Chang and Qiu (64)	2 male (Roger Federer, Rafael Nadal)	RF = 26–38 RN = 21–33	HP (*n* = 2)	Development of an expert system for decision making in tennis matches, in which tennis coaches evaluate the tactical-technical skills of tennis players and a classification model was developed using notation analysis and the decision tree algorithm.	Classification accuracy of the C5.0 algorithm > Qiu's method Highest scored strokes: baseline forehand and backhand, midcourt backhand
Cowden (65)	43; male = 25, female = 18	13.6 ± 2.4	N (12-and-under = 8; 14-and-under = 18; 16-and-under = 8, older = 9)	Participants completed the mental toughness (MT) inventory and the scores recorded during a tennis match were used to create performance indices for each athlete.	MT significantly correlated with ranking, match outcome, % of three or more consecutive points won per game, ratio of break points, % of points won when scores were level on serve and return, % of serve points won when ahead, % of return points won when behind.
Dimic et al. (66)	2 male (Roger Federer, Novak Djokovic)	RF = 38 ND = 32	HP (*n* = 2)	Analyze how the efficiency and frequency of disguised (DIS) and undisguised (UND) strokes effect on players in rallies.	Proportion of disguised strokes: RF > ND Winning a point: DIS > UND incorrect reactions of player: DIS > UND
Féry and Crognier (67)	7 male	24.8	A (*n* = 7)	Monitor anticipatory cues to accurately estimate the spatio-temporal characteristics of the 4 different trajectories of incoming balls in 3 tactical situations.	Error: Video < *in situ*ation Confidence estimations ≠ temporal estimations
Fitzpatrick et al. (68)	48 male	MTR = 7.4 ± 0.6; MTO = 8.5 ± 0.6, MTG = 9.9 ± 0.4; FB = 13.7 ± 0.5	MTR (*n* = 18); MTO (*n* = 16); MTG (*n* = 8); FB (*n* = 6)	Determining the influence of court size scaling and ball modification tasks on the development of playing behavior in young tennis players on red (MTR), orange (MTO), green (MTG) and full court (FB).	Rally length: MTR > MTG, FB % forehands: MTR > MTO, MTG, FB % backhands: MTR < MT, MTG, FB % net play: MTR < FB % errors: MTR < FB % first serve: MTR > MTO, MTG % Aces: MTG > MTR, MTO, FB
Garcia-Gonzalez et al. (69)	12 male	HP = 16.1 ± 2.3; A = 16.3 ± 2.3	HP (*n* = 6), A (*n* = 6)	Evaluation of differences in tactical knowledge between high-performance players (HP) and advanced players (A) based on oral reports.	tactical knowledge: HP > A complex structures: HP > A long-term memory: HP > A
García-González et al. (70)	11 male	CG = 12.83 ± 0.75 EG = 13.00 ± 0.71	CG = 6 (3.67 ± 0.52) EG = 5 (3.60 ± 0.54)	Evaluation of the effects of a decision training program on the decision-making ability and performance of tennis players (CG—control group; EG—experimental group).	% successful decisions: *EG *> CG execution skills: *EG *> CG
Gimenez-Egido et al. (71)	20 male	9.46 ± 0.66	Tennis experience = 3.65 ± 1.53	Analysis of tennis players’ serve performance in two tournament formats (GC—green, modified court—MC).	% first serve success: MC > GC % aces: MC > GC % unreturned serves: MC > GC % net errors: GC > MC
Kolman et al. (72)	32 male	13.4 ± 0.5	Elite (*n* = 15)–Sub-elite (*n* = 17)	Development of a reliable, valid and feasible technical-tactical test that can be used to identify and promote talent in tennis.	velocity- accuracy-index: E > SE mean velocity: E > SE mean accuracy: E > SE % errors: E < SE
Kovalchik and Reid (73)	33 male–27 female	HP = 23.0–24.2 J = 17.1–17.5	HP (*n* = 42) J (*n* = 18)	Description of the age and performance profile of elite junior tennis players, comparison of match, game and stroke characteristics and comparison of the match factors most strongly associated with match wins in junior (J) and professional tennis (HP).	Rally length: HP > J Shot speed: HP > J Speed at baseline: HP > J Net clearance: HP > J Sideline distance (FH): HP* *< J Sideline distance (BH): HP* *< J Distance from baseline (FH): HP > J Distance from baseline (BH): HP < J
Raschke and Lames (74)	24 male	10–14	CG = 12 EG = 12	Development of a video tactics training program for young top tennis players and testing of its effectiveness.	Serving: EG > CG Receiving: EG > CG–Baseline game: EG > CG Preparation & finish: EG > CG

RF, Roger Federer; RN, Rafael Nadal; ND, Novak Djokovic; HP, high performance player; A, advanced player; N, national level player; J, junior; CG, control group, EG, experimental group; MTR, red court/ball; MTO, orange court/ball; MTG, green court/ball; FB, full court/normal ball; FH, forehand; BH, backhand; CI, contextual interference; SA, situation awareness; ANT, anticipation; DM, decision making; MT, mental toughness; DIS, disguised stroke; UND, undisguised strokes.

In the articles selected, the main topics studied included the following: technical-tactical skills, game situations, and match performance. The results will be presented according to the different studies grouped according to these main topics.

### Technical-tactical skills

Eight studies analysed tactical-technical skills (62, 63, 66, 68–70, 72, 74). Five of these studies were of high methodological quality, and three studies were of good methodological quality.

The contextual interference (CI) effect is a well-established phenomenon in motor learning and was discussed in an article of Buszard et al. (62) that was split into two studies. In the first study, video clips were used to observe the type of tennis stroke, the side of the serve and the placement of the serve. They differentiated between-skill and within-skill variability during practice. No differences in between-skill variability were observed in young tennis players during serve training. Within-skill variability was greater for the placement of the serve than for the side from which the young tennis players served. No significant correlations were found between serve accuracy and between-skill or within-skill variability.

In the second study, the effect of CI on serve learning was examined under “blocked” (low CI) and “serial” (moderate CI) practice conditions. The percentage of serves that reached the target was higher in moderate CI than in low CI, there were no differences in the percentage of successful serves and in serve velocity, while serve displacement was lower in the low CI group and serves were directed towards T (skill test) in the post-test.

The effectiveness of tennis training instructions on situational awareness (SA), anticipation (A) and decision-making (DM) was investigated by Caserta and Singer (63). After receiving instructions, participants responded to a series of edited video clips. The results suggested that combined SA, A, and DM awareness training is effective in improving performance. They concluded that no difference was found between implicit and explicit learning strategies.

In another study, the importance of decision-making for the performance of tennis players was explored by García-González et al. (70). The decision training programme was based on an analysis that combined tactical questions and video feedback on the players’ own actions. The experimental group significantly improved decision making and performance and maintained these improvements during the retention phase. The authors concluded that video feedback in combination with questions can be recommended to improve tactical decision making in tennis.

Dimic et al. (66) analysed the less discussed area of anticipation, more specifically the influence of stroke intentions and “disguise” on a player's performance in a match. Disguised strokes resulted in a significantly higher proportion of incorrect responses by receivers compared to undisguised strokes and are significantly more effective than undisguised strokes in gaining a point or advantageous position. The authors suggest that this area should be included in the technical-tactical training of junior players.

The differences in the tactical knowledge of novice and entry professional tennis players were investigated by Garcia-Gonzalez et al. (69). They used the protocol of McPherson and Thomas to analyse the verbal reports during the game. They found significant differences in terms of conceptual content, structure, and sophistication. Entry professional tennis players had greater, more sophisticated, and differentiated tactical knowledge; more complex structures are developed in long-term memory with increasing experience. Specific tactics training can improve the tactical knowledge and cognitive skills of tennis players.

The study by Kolman et al. (72) is one of the few to have investigated the reliability, validity, and feasibility of an instrument for the integrated tactical-technical analysis of tennis players. They studied young tennis players at elite I and sub-elite (SE) level and found that tennis players at elite level had a better speed-accuracy index, higher average speed and accuracy and a lower percentage of errors in their game than tennis players at sub-elite level. They concluded that the test instrument has reasonable reliability and validity and is of practical value to tennis coaches.

The latest article by Fitzpatrick et al. (68) investigated how court size and ball type affect the performance of young tennis players. The results showed several specific adaptations to playing characteristics on different courts and balls (MR—red, MO—orange, MG—green, B—normal). MTR rallies were longer than FB ball rallies, and a higher percentage of forehands were played. The percentage of successful first serves was higher on MTR than on the other courts. The percentage of errors, double faults and net play was lower on MTR than on the other courts. The results suggest that a gradual transition from smaller courts to full-size courts and the use of appropriate balls is necessary for the optimal development of younger tennis players’ game.

### Game situations

Two studies investigated game situations in tennis matches, and both were of good methodological quality. The first study (64) analysed the matches of two elite tennis players based on the assessment of tennis coaches and established a classification model that incorporated the players’ playing status, hitting habits and tennis skills, which formed the basis for the matches in which the notation analysis and decision tree algorithm were used. The study proved that integrated analysis of tennis games at the highest level is possible with the help of modern technologies, which can be used for imaginary training of other players so that players can gain experience through continuous simulation of training and game analysis.

The second study (67) investigated perceptual anticipation in realistic, meaningful situations and showed that the essential anticipatory information is contained in the view of the opponent's stroke movements, regardless of the tactical significance of the situation. The authors recommend a comprehensive analysis of tennis play and more ecological test conditions. However, no statistical tests were conducted to confirm these visible differences.

### Match performance

Three studies analysed match performance in tennis. One of these studies was of high methodological quality, and two studies were of good methodological quality. Kovalchik & Reid (73) analysed a large number of match and stroke performance indicators of elite junior players during matches and determined the effects on performance in professional and junior tennis. Professional players had a greater serve advantage, professional men and women won more points on serve as a percentage, converted more break points, and executed more powerful and accurate shots than juniors. Professional men did 50% more work overall in the game than juniors, and junior women did 50% more work than professional women. The conclusions showed that the demands of the game and the physical characteristics of the shots differ between juniors and professionals.

The playing characteristics and indicators of serve performance of young tennis players playing with green and orange balls and on a suitable court were compared by Gimenez-Egido et al. (71). The conditions set by the young tennis players (court size, net height, ball type) have a significant influence on the observed game characteristics (e.g., length of rallies) and performance indicators (e.g., number of aces). Two studies investigated the psychological characteristics of a tennis game under competitive conditions.

In their study, Cowden (65) investigated the relationship between mental strength and performance measures in tennis. As expected, the results show a positive influence of mental strength on successful performance in competitive tennis (e.g., ranking, match outcome, follow-up points won,…), especially in certain match situations. Only in this study were statistical tests performed to confirm a significant correlation.

## Discussion

The aim of this review was to provide an overview of the research on technical and tactical competencies to define the different levels of play in the game of tennis. The intention was to explore the possibilities that the competencies and the competency model approach offer for the description, identification and differentiation of tennis playing levels and, eventually, to pave the way for the creation of a model framework that would assist in the further development of a competency-based model applied to tennis. Not studies have been found that comprehensively address tactical effectiveness and technical efficiency and analyse the level of play or the performance of tennis players by their competence as a combination of skills and knowledge application. In this review, a number of studies have been found in which skills are used instead of competences, which is a subordinate concept. As indicated above, the term competence includes skills, behaviours, knowledge, and abilities that enable a player to perform certain actions effectively in sport.

Competency-based models have also been used in areas other than analysing athlete performance in the study of Fletcher and Maher (75), in which they developed a competency model and its implications for applied sport psychology and professional development and lifelong learning in applied sport psychology. For the sport management, Toh and Jamieson (76) construct and validate a survey instrument to determine and develop of sport management competencies and model. The analysis of coaches’ competencies in team sports in four areas (motivation, game strategy, technique and character-building competencies) showed that improving coaches’ psychological and tactical skills and their ability to recognize skills, together with a positive attitude towards sport, can help to improve athletes’ confidence in the work of coaches (77). Lim et al. (78) found that the competence levels of coaches did not differ significantly by gender and performance of student athletes, but did differ significantly by sport type, between team and individual sports. The competency model proved to be a useful tool for sports organizations to identify and recruit coaches and for coaches to apply coaching planning strategies to ensure the effectiveness of their work. In reviewing the literature, we have not found any articles that would design a competency model to determine the level of play, important skills, and performance indicators in tennis, or we have only found attempts to design a model in a narrower field of research.

Studies analysing inter-skill variability during practice found that skilled tennis players generally perform serve drills with little contextual interference. The differences were influenced by the direction of the serve (placement) and the side from which the serve was executed, but no differences were found in terms of percentage of hits, displacement of the serve and speed (62). Whiteside et al. (79) concluded that there are no differences in body kinematics between successful serves and serve faults. Serves into the net are characterized by projection angles that are significantly further below the horizontal. They conclude that the coordination of the distal degrees of freedom and a sophisticated coupling of perception and action appear to be more important for success than any isolated mechanical component of the serve. Other conclusions were reached by Antunez et al. (80), who found that an increase in movement variability can have a negative effect on serve performance in tennis by reducing the speed and accuracy of the ball. Meanwhile, Whiteside et al. (81) investigated the coordinated joint rotations and variability of the lower limbs, trunk, service arm and ball position during the serve of elite female tennis players. The coordinated joint rotations of the lower limbs and trunk appeared to be most consistent at the time the players left the ground. The variability in the two degrees of freedom of the elbow became significantly greater as the serve approached. Despite the variable ball throw, the temporal composition of the serve was very consistent, supporting previous claims that players use the position of the ball to regulate their movement.

Two studies investigated the effectiveness of a decision training program and specific instructions on decision making and performance in tennis. They found positive effects of the program on game performance, reaction speed, accuracy (63) and the percentage of successful decisions (70). García-Gonzalez et al. (82) found in an earlier study a positive characteristic correlation between tennis players’ knowledge, decision-making during the serve and the decision-making aspects that tennis players develop during the game. Decision making in tennis is also closely linked to cognitive perceptual abilities, including anticipation. Caserta et al. (83) confirmed that training of perceptual-cognitive skills resulted in significantly faster reaction time, a higher percentage of accurate responses and a higher percentage of performance decisions. García et al. (84) showed differences in tactical knowledge, which is more structured and sophisticated than the declarative and procedural level of elite tennis players.

The model of decision training developed by Vickers (85) explains the building of tactical experiences that promote the development of tactical knowledge and cognitive skills, including the training of cognitive skills related to decision making. The mentioned model was used by García-González et al. (70) and confirmed the usefulness of a combination of different tools such as video feedback or questions as explicit learning methods and can achieve improvements for highly complex situations. By applying the decision training model, tennis players were able to significantly improve their decision making and game performance and maintain these improvements during the retention phase. Vernon et al. (86) investigated the individual contribution of kinematic or contextual information sources to the anticipation ability of an experienced athlete in a time pressure situation, when returning a serve. They used a qualitative interview methodology to investigate this interaction. The results support previous work that has independently examined the different anticipatory sources of information available to a tennis player and the fact that players weight or prioritize certain information under certain circumstances. Otherwise, essential anticipatory information, regardless of the tactical significance of the situation, is contained in the view of the opponent's stroke movements (67). Garcia-Gonzalez et al. (69) investigated tactical knowledge, specifically the problem of strategic planning and found significant differences in conceptual content, complex structure, long-term memory and sophistication between professional and advanced tennis players. Raschke and Lames (74) also used the method of video-based tactics training and confirmed significant improvements in cognitive abilities in the interpretation of tactical behaviour in practice and matches. As shown in previous studies, specific training programs can improve the tactical knowledge and level of cognitive skills of tennis players by creating a relevant knowledge base that increases with experience and expertise. The inclusion of specific program to improve tactical skills and decision making should be present at different stages of skill development so that significant improvements in cognitive, behavioural, and performance-related variables could occur.

Athletes’ anticipation is based on contextual cues resulting from the opponent's movement towards the strike position and from the opponent's posture prior to impact (87). Advanced players can pay attention to these cues and obtain detailed and reliable information about when to expect a hit. On the other hand, they tend to “disguise” their intention to hit, which confuses their opponent's assessment and impairs their anticipatory reaction. Hiding the intention is a very specific skill that allows for greater unpredictability at the level of top tennis players and at the same time creates a tactical advantage for the player and a disadvantage for the opponent (66).

In tennis, great importance is given to psychological aspects, especially at the higher performance level. One component of mental strength that is particularly relevant in sport, especially in tennis, is the control of thoughts and emotions. In reviewing the literature, we excluded only one study from the psychological domain, which certainly does not reflect the importance, but rather the complexity of the domain and the difficulty of analysing it under competitive conditions. Cowden (65) notes that mental toughness appears to depend on the nature of the performance area and the context of the game and that it is impossible to address this area independently of the physical, technical, tactical and psychological characteristics of athlete and opponent.

As per the practical application of this study, the tools identified in this systematic review are important for analysing how coaching and competition conditions can be adapted for young athletes. Designing effective sport tasks and competitions for young athletes is a complex process in which many factors interact in the learning of functional skills and behaviours. Reducing the height of the net and the size of the court improves serving performance and thus creates a useful learning environment for young tennis players (71). Adapted training and playing conditions for young tennis players influence many performance indicators such as the length of rallies, the variety of strokes and the success of the serve (68), the number of winners, forced errors, volleys played, the height of the stroke impact zone and the player's position on the court (88), as well as the style of play (89).

In match play scenarios, for the performance or notation analysis of the tennis game, in addition to the already established visual or video analysis, artificial intelligence methods have recently been used more frequently. These methods enable the analysis of a large amount of data on the players’ stroke and movement techniques, the trajectories and landing points of the balls and the general observation of both tennis players during the match. Chang and Qiu (64) analysed two elite tennis players and, together with tennis experts, developed a decision tree algorithm that took into account game results, hitting habits and tennis knowledge. The use of this decision-making system is a useful tool for comprehensive and detailed analysis as well as a training tool for tennis players to gain experience. The tactical-technical instrument, analyses tennis players in different tactical situations (i.e., offensive, neutral and defensive), is a reliable, valid and practicable test that, in addition to analysis, enables the identification of talent and the long-term development of young tennis players (72). An equally valid and reliable tool for monitoring the content of technical-tactical training sessions on courts was developed by Penalva et al. (90), which allows tennis coaches to improve the planning and programming of on-court training in order to contribute more efficiently to the development of players, regardless of their level of play.

It is therefore realistic to use artificial intelligence methods based on the mining of event sequences to make it possible to summarize a large number of different patterns, constant interdependencies in the performance of both athletes, multivariate strokes and movements, and positions on the court, which are common to all racket sports (91).

Therefore, the application of a competency model in tennis is possible if an integrated approach is chosen for the problem to be addressed and an appropriate amount, accuracy and depth of data is available. Currently, this is only possible with automatic motion tracking systems such as Hawk-Eye. With the system it is possible to identify the similarities and differences in the stroke and movement characteristics of different groups of tennis players (i.e., men, women, professionals, juniors). Kovalchik and Reid (73) with the method of data collection, the selection of performance indicators and the methods used to analyse and present the data comes closest to a holistic view of match play. Such an approach is the basis for the creation of a competency model and thus also a tool to understand how competitiveness, playing demands and physical characteristics of strokes differ between quality groups of tennis players.

There are several limitations of this study that can be mentioned. First, a methodology based on Law et al. (61) was used for this systematic review, in which two experts independently rated the quality of the selected articles. Since the experts’ ratings differed slightly, the average scores were calculated. Secondly, there are several technical limitations in the comprehensive analysis of tennis when addressing several important areas simultaneously. The technical limitations of collecting data on players during matches influence the researchers’ decision to choose only a narrower range of observations to analyse the game. All this leads to a lack of studies examining tactical and technical skills/competencies, psychological functioning, and fitness during a tennis match. With the advent of modern technologies, machine learning and artificial intelligence, the possibilities for a comprehensive analysis of tennis play during the match and for determining the individual quality level of tennis players will increase. At the same time, tennis experts must use modern technologies to create theoretical models that form the basis for testing skill-based models. Only in this way will it be possible to transfer theoretical knowledge into practice.

## Conclusions

As it has been stated, success in tennis is a multifaceted concept that transcends mere victory on the court; it encompasses personal growth, achievement of individual goals, and recognition within the broader tennis community.

The number of articles dealing with tennis under real conditions and linking individual narrower areas of research (such as tactics and technique) is currently quite small. In addition to analyzing individual factors that influence the efficiency and effectiveness of tennis players, it is necessary to look for interactions between individual areas and factors. It is precisely the search for reciprocal relationships between factors that enables a more precise insight into the tennis game and the performance of tennis players. In addition, the use of competency models enables the analysis of complex sports such as tennis.

The complexity and dynamics of the game of tennis should not be an obstacle to comprehensive research and application of the competency model in this area. Future lines of research could consider the creation of competency models of tennis levels of play that could include, at least, three key elements: (1) key competencies, (2) description of standards, (3) evidence. The development of tennis competency models for different quality and age groups, both genders and individual game situations must initially take place at the level of evidence or performance indicators. In the following, the models must be supplemented by a description of standards and key competencies for the tennis player groups mentioned. There are already some attempts to determine the competencies for different age and quality levels of tennis players, but only at expert level and they have not been published and verified with scientific methods. For a better understanding, an example of the competence of a young female tennis player is provided: “When playing at the baseline, the player shows that she is able to play appropriate strokes depending on the opponent's stroke quality and taking into account her placement.” Similarly, in the future it will be necessary to create theoretical competence models and test them using both scientific methods and practical tests.

## Data Availability

The raw data supporting the conclusions of this article will be made available by the authors, without undue reservation.

## References

[1] Cui DY. Exploring Match Performance of Elite Tennis Players: The Multifactorial Game-Related Effects in Grand Slams. Madrid: Universidad Politécnica De Madrid (2018).

[2] Torres-Luque G Fernández-García ÁI Cabello-Manrique D Giménez-Egido JM Ortega-Toro E. Design and validation of an observational instrument for the technical-tactical actions in singles tennis. Front Psychol. (2018) 9:1–10. 10.3389/fpsyg.2018.02418 30559702 PMC6287015

[3] Michel MF Girard O Guillard V Brechbuhl C. Well-being as a performance pillar: a holistic approach for monitoring tennis players. Front Sports Act Living. (2023) 5:1259821. 10.3389/fspor.2023.1259821 37789864 PMC10544573

[4] Newton M Duda JL. Elite adolescent athletes’ achievement goals and beliefs concerning success in tennis. J Sport Exerc Psychol. (1993) 15(4):437–48. 10.1123/jsep.15.4.437

[5] Elliott B. Biomechanics and tennis. Br J Sports Med. (2006) 40:392–6. 10.1136/bjsm.2005.023150 16632567 PMC2577481

[6] Klaus A Bradshaw R Young W O’Brien B Zois J. Success in national level junior tennis: tactical perspectives. Int J Sports Sci Coach. (2017) 12(5):618–22. 10.1177/1747954117727792

[7] Pankhurst A. 10u tennis: the essentials of developing players for the future. In: Colvin AC Gladstone JN, editors. The Young Tennis Player: Injury Prevention and Treatment. Cham: Springer (2016). p. 1–16.

[8] Giles B Peeling P Dawson B Reid M. How do professional tennis players move? The perceptions of coaches and strength and conditioning experts. J Sports Sci. (2019) 37(7):726–34. 10.1080/02640414.2018.1523034 30319029

[9] Brouwers J De Bosscher V Sotiriadou P. An examination of the importance of performances in youth and junior competition as an indicator of later success in tennis. Sport Management Review. (2012) 15(4):461–75. 10.1016/j.smr.2012.05.002

[10] De Bosscher V De Knop P Heyndels B. Comparing tennis success among countries. Int Sports Stud. (2003) 25(1):49–68.

[11] Filipcic A Panjan A Reid M Crespo M Sarabon N. Tournament structure and success of players based on location in men’s professional tennis. J Sports Sci Med. (2013) 12(2):354–61. 24149816 PMC3761828

[12] Turner M Ishihara T Beranek P Turner K Fransen J Born P Investigating the role of age and maturation on the association between tennis experience and cognitive function in junior beginner to intermediate-level tennis players. Int J Sports Sci Coach. (2022) 17(5):1071–8. 10.1177/17479541211055841

[13] Reid M Crespo M Lay B Berry J. Skill acquisition in tennis: research and current practice. J Sci Med Sport. (2007) 10(1):1–10. 10.1016/j.jsams.2006.05.011 16809063

[14] Perri T Duffield R Murphy A Mabon T McGillivray I Reid M. Macro periodisation of competition in international women’s tennis: insights for long-term athlete development. Int J Sports Sci Coach. (2023) 9:788–96. 10.1177/17479541231171695

[15] Bane MK Reid M Morgan S. Has player development in men’s tennis really changed? An historical rankings perspective. J Sports Sci. (2014) 32(15):1477–84. 10.1080/02640414.2014.899706 24750027

[16] Shrom SJ Cumming J Fenton S-J. Lifestyle challenges and mental health of professional tennis players: an exploratory case study. Int J Sport Exerc Psychol. (2022) 21(6):1070–90. 10.1080/1612197X.2022.2099947

[17] Fernández-García ÁI Blanca-Torres JC Nikolaidis PT Torres-Luque G. Differences in competition statistics between winners and losers in male and female tennis players in Olympic games. Germ J Exerc Sport Res. (2019) 49(3):313–8. 10.1007/s12662-019-00608-y

[18] Fuentes-Garcia JP Villafaina S Martinez-Gallego R Crespo M. Pre- and post-competitive anxiety and match outcome in elite international junior tennis players. Int J Sports Sci Coach. (2022) 9:2108–16. 10.1177/17479541221122396

[19] Ulbricht A Fernandez-Fernandez J Mendez-Villanueva A Ferrauti A. Impact of fitness characteristics on tennis performance in elite junior tennis players. J Strength Cond Res. (2016) 30(4):989–98. 10.1519/JSC.0000000000001267 26605803

[20] Buscombe R Greenlees I Holder T Thelwell R Rimmer M. Expectancy effects in tennis: the impact of opponents’ pre-match non-verbal behaviour on male tennis players. J Sports Sci. (2006) 24(12):1265–72. 10.1080/02640410600598281 17101528

[21] Torres-Luque G Fernandez-Garcia AI Sánchez-Pay A Ramírez A Nikolaidis PT. Differences in the statistics of competition in individual tennis according to the playing surface in male junior players of high level. Revista Euroamericana Ciencias Deporte. (2017) 6:75–80. 10.6018/280431

[22] Bergeron MF. Youth sports in the heat recovery and scheduling considerations for tournament play. Sports Med. (2009) 39(7):513–22. 10.2165/00007256-200939070-00001 19530749

[23] Dube SK Tatz SJ. Audience effects in tennis performance. Percept Mot Skills. (1991) 73(3):844–6. 10.2466/pms.1991.73.3.844

[24] Baron MP Sasseville N Manon D Bizot D. Development of professional competencies. Int J Interdiscipl Educ Stud. (2019) 14(1):79. 10.18848/2327-011X/CGP/v14i01/79-93

[25] Matic RM Gonzalez-Serrano MH Damnjanović J Maksimovic B Papić-Blagojević N Milošević I Professional competencies development of sports science students: the need for more entrepreneurship education. Manag Mark Challenges Knowledge Soc. (2022) 17(s1):426–48. 10.2478/mmcks-2022-0024

[26] Siedentop D Hastie P van der Mars H. Complete Guide to Sport Education. Champaign, IL: Human Kinetics (2011).

[27] Collins D Burke V Martindale A Cruickshank A. The illusion of competency versus the desirability of expertise: seeking a common standard for support professions in sport. Sports Med. (2015) 45(1):1–7. 10.1007/s40279-014-0251-1 25208494

[28] Kolman NS Kramer T Elferink-Gemser MT Huijgen BCH Visscher C. Technical and tactical skills related to performance levels in tennis: a systematic review. J Sports Sci. (2019) 37(1):108–21. 10.1080/02640414.2018.1483699 29889615

[29] Davies K Roeter P E. View of a business perspective of the United States tennis association’s American development model. Coach Sport Sci Rev. (2023) 30(90):13–6. 10.52383/itfcoaching.v31i90.456

[30] Zmajic H. Los sistemas de formación de entrenadores en Europa: comparación de las competencias. ITF Coach Sport Sci Rev. (2011) 19(54):10–3. 10.52383/itfcoaching.v19i54.534

[31] Kinnerk P Harvey S MacDonncha C Lyons M. A review of the game-based approaches to coaching literature in competitive team sport settings. Quest. (2018) 70(4):401–18. 10.1080/00336297.2018.1439390

[32] Crespo M McInerney P Reid M. Long term coach development. ITF Coach Sport Sci Rev. (2006) 14(40):2–4.

[33] Campos J Sebastiani EM Crespo M. The professional competences of the tennis coach. The vision of their tutors at international level. Apunts Educ Física Deportes. 2017(129):138. 10.5672/apunts.2014-0983.es.(2017/3).129.10

[34] Takahashi H. Performance analysis in tennis since 2000: a systematic review focused on the methods of data collection. Int J Racket Sports Sci. (2022) 4(2):40–55. 10.30827/digibug.80900

[35] Hadžić V Germič A Filipcic A. Validity and reliability of a novel monitoring sensor for the quantification of the hitting load in tennis. PLoS One. (2021) 16(7):1–13. 10.1371/journal.pone.0255339 34324580 PMC8321100

[36] Cross R Pollard G. Grand slam men’s singles tennis 1991–2009 serve speeds and other related data. ITF Coach Sport Sci Rev 2009. (2009) 16(49):8–10.

[37] Cui YX Gomez MA Goncalves B Liu HY Sampaio J. Effects of experience and relative quality in tennis match performance during four grand slams. Int J Perform Anal Sport. (2017) 17(5):783–801. 10.1080/24748668.2017.1399325

[38] Hizan H Whipp P Reid M. Gender differences in the spatial distributions of the tennis serve. Int J Sports Sci Coach. (2015) 10(1):87–96. 10.1260/1747-9541.10.1.87

[39] Fernandez-Garcia AI Torres-Luque G Sanchez-Pay A Cabello-Manrique D, editors. Differences in game statistics between men and junior boys in different surfaces. 14th International Table Tennis Sports Science Congress/5th World Congress of Science and Rocket Sports; 2015 Apr 23–25; Int Table Tennis Federat, Suzhou, PEOPLES R CHINA. Suzhou: Soochow Univ Press (2017). p. 114–9.

[40] Fitzpatrick A Stone JA Choppin S Kelley J. A simple new method for identifying performance characteristics associated with success in elite tennis. Int J Sports Sci Coach. (2018) 14(1):43–50. 10.1177/1747954118809089

[41] Wei X Lucey P Morgan S Sridharan S. Predicting shot locations in tennis using spatiotemporal data. 2013 International Conference on Digital Image Computing: Techniques and Applications (DICTA) (2013). p. 1–8

[42] Stare M Zibrat U Filipcic A. Stroke effectivness in professional and junior tennis. Kinesiol Slovenica. (2015) 21(2):39–50.

[43] Martin C Bideau B Touzard P Kulpa R. Identification of serve pacing strategies during five-set tennis matches. Int J Sports Sci Coach. (2018) 14(1):32–42. 10.1177/1747954118806682

[44] Reid M Morgan S Whiteside D. Matchplay characteristics of grand slam tennis: implications for training and conditioning. J Sports Sci. (2016) 34(19):1791–8. 10.1080/02640414.2016.1139161 27009823

[45] Whiteside D Reid M. External match workloads during the first week of Australian open tennis competition. Int J Sports Physiol Perform. (2017) 12(6):756–63. 10.1123/ijspp.2016-0259 27834557

[46] Whiteside D Reid M. Spatial characteristics of professional tennis serves with implications for serving aces: a machine learning approach. J Sports Sci. (2017) 35(7):648–54. 10.1080/02640414.2016.1183805 27189847

[47] Carboch J Sklenářik M Kočíb T Zháněl J. Game characteristics in professional tennis at different levels of international tournaments. Int J Appl Exerc Physiol. (2021) 10(1):129–37.

[48] Kovalchik S Reid M. A shot taxonomy in the era of tracking data in professional tennis. J Sports Sci. (2018) 36(18):2096–104. 10.1080/02640414.2018.1438094 29419342

[49] Ishihara T Kuroda Y Mizuno M. Competitive achievement may be predicted by executive functions in junior tennis players: an 18-month follow-up study. J Sports Sci. (2019) 37(7):755–61. 10.1080/02640414.2018.1524738 30332916

[50] Gould D Lauer L Rolo C Jannes C Pennisi N. Understanding the role parents play in tennis success: a national survey of junior tennis coaches. Br J Sports Med. (2006) 40(7):632–6. discussion 6. 10.1136/bjsm.2005.024927 16702176 PMC2564313

[51] Pummell EKL Lavallee D. Preparing UK tennis academy players for the junior-to-senior transition: development, implementation, and evaluation of an intervention program. Psychol Sport Exerc. (2019) 40:156–64. 10.1016/j.psychsport.2018.07.007

[52] Li P De Bosscher V Weissensteiner JR. The journey to elite success: a thirty-year longitudinal study of the career trajectories of top professional tennis players. Int J Perform Anal Sport. (2018) 18(6):961–72. 10.1080/24748668.2018.1534197

[53] Reid M Morgan S Churchill T Bane MK. Rankings in professional men’s tennis: a rich but underutilized source of information. J Sports Sci. (2014) 32(10):986–92. 10.1080/02640414.2013.876086 24506799

[54] Reid M McMurtrie D Crespo M. The relationship between match statistics and top 100 ranking in professional men’s tennis. Int J Perform Anal Sport. (2010) 10(2):131–8. 10.1080/24748668.2010.11868509

[55] Whiteside D Bane M Reid M. Differentiating top-ranked male tennis players from lower-ranked players using hawk-eye data: an investigation of the 2012–2014 autralian open tournaments. 33rd International Conference on Biomechanics in Sports. Poitiers, France (2015).

[56] Ma SM Liu CC Tan Y Ma SC. Winning matches in grand slam men’s singles: an analysis of player performance-related variables from 1991 to 2008. J Sports Sci. (2013) 31(11):1147–55. 10.1080/02640414.2013.775472 23458278

[57] Filipcic A Leskosek B Crespo M Filipcic T. Matchplay characteristics and performance indicators of male junior and entry professional tennis players. Int J Sports Sci Coach. (2021) 16(3):768–76. 10.1177/1747954120988002

[58] O’Donoghue P Cullinane A. A regression-based approach to interpreting sports performance. Int J Perform Anal Sport. (2017) 11(2):295–307. 10.1080/24748668.2011.11868549

[59] Page MJ McKenzie JE Bossuyt PM Boutron I Hoffmann TC Mulrow CD The PRISMA 2020 statement: an updated guideline for reporting systematic reviews. Br Med J. (2021) 372:n71. 10.1136/bmj.n71 33782057 PMC8005924

[60] Amir-Behghadami M Population JA Intervention C. Outcomes and study (PICOS) design as a framework to formulate eligibility criteria in systematic reviews. Emerg Med J. (2020) 37(6):387. 10.1136/emermed-2020-209567 32253195

[61] Law M Stewart D Letts L Pollock N Bosch J Westmorland M. Guidelines for Critical Review Form-Qualitative Studies. Hamilton: McMaster University (1998).

[62] Buszard T Reid M Krause L Kovalchik S Farrow D. Quantifying contextual interference and its effect on skill transfer in skilled youth tennis players. Front Psychol. (2017) 8:1931. 10.3389/fpsyg.2017.01931 29163306 PMC5676081

[63] Caserta RJ Singer RN. The effectiveness of situational awareness learning in response to video tennis match situations. J Appl Sport Psychol. (2007) 19(2):125–41. 10.1080/10413200601184712

[64] Chang C-W Qiu Y-R. Constructing a gaming model for professional tennis players using the C5.0 algorithm. Appl Sci. (2022) 12(16):8222. 10.3390/app12168222

[65] Cowden RG. Competitive performance correlates of mental toughness in tennis: a preliminary analysis. Percept Mot Skills. (2016) 123(1):341–60. 10.1177/0031512516659902 27502244

[66] Dimic M Furuya R Kanosue K. Importance of disguising groundstrokes in a match between two top tennis players (Federer and Djokovic). Int J Sports Sci Coach. (2022) 18(1):257–69. 10.1177/17479541221075728

[67] Féry Ya Crognier L. On the tactical significance of game situations in anticipating ball trajectories in tennis. Res Q Exerc Sport. (2001) 72(2):143–9. 10.1080/02701367.2001.10608944 11393877

[68] Fitzpatrick A Davids K Stone JA. Effects of lawn tennis association mini tennis as task constraints on children’s match-play characteristics. J Sports Sci. (2017) 35(22):1–7. 10.1080/02640414.2016.1261179 27905867

[69] Garcia-Gonzalez L Moreno A Moreno MP Iglesias D Del Villar F. Tactical knowledge in tennis: a comparison of two groups with different levels of expertise. Percept Mot Skills. (2012) 115(2):567–80. 10.2466/30.10.25.PMS.115.5.567-580 23265019

[70] García-González L Moreno A Gil A Moreno MP Villar FD. Effects of decision training on decision making and performance in young tennis players: an applied research. J Appl Sport Psychol. (2014) 26(4):426–40. 10.1080/10413200.2014.917441

[71] Gimenez-Egido JM Ortega-Toro E Palao JM Torres-Luque G. Effect of scaling equipment on U-10 players tennis serve during match-play: a nonlinear pedagogical approach. Chaos, Solitons Fractals. (2020) 139:110011. 10.1016/j.chaos.2020.110011

[72] Kolman N Huijgen B Kramer T Elferink-Gemser M Visscher C. The Dutch technical-tactical tennis test (D4 T) for talent identification and development: psychometric characteristics. J Hum Kinet. (2017) 55:127–38. 10.1515/hukin-2017-0012 28210345 PMC5304281

[73] Kovalchik SA Reid M. Comparing matchplay characteristics and physical demands of junior and professional tennis athletes in the era of big data. J Sports Sci Med. (2017) 16:489–97. 29238248 PMC5721178

[74] Raschke A Lames M. Video-based tactic training in tennis proof of efficacy in a field experiment with 10-to 14-year-old tournament players. Germ J Exerc Sport Res. (2019) 49(3):345–50. 10.1007/s12662-019-00598-x

[75] Fletcher D Maher J. Toward a competency-based understanding of the training and development of applied sport psychologists. Sport Exerc Perform Psychol. (2013) 2(4):265–80. 10.1037/a0031976

[76] Toh KL Jamieson LM. Constructing and validating competencies of sport managers (COSM) instrument: a model development. Recreat Sports J. (2000) 24(2):38–55. 10.1123/nirsa.24.2.38

[77] Kao S-F Hsieh M-H Lee P-L. Coaching competency and trust in coach in sport teams. Int J Sports Sci Coach. (2017) 12(3):319–27. 10.1177/1747954117710508

[78] Lim CK Mahat NI Marzuki NA Khor HP. Student-athletes’ evaluation of coaches’ coaching competencies and their sport achievement motivation. Rev Eur Stud. (2014) 6(2):17–30. 10.5539/res.v6n2p17

[79] Whiteside D Elliott B Lay B Reid M. A kinematic comparison of successful and unsuccessful tennis serves across the elite development pathway. Hum Mov Sci. (2013) 32(4):822–35. 10.1016/j.humov.2013.06.003 23973088

[80] Antunez RM Hernandez FJ Garcia JP Vaillo RR Arroyo JS. Relationship between motor variability, accuracy, and ball speed in the tennis serve. J Hum Kinet. (2012) 33:45–53. 10.2478/v10078-012-0043-3 23486998 PMC3588677

[81] Whiteside D Elliott BC Lay B Reid M. Coordination and variability in the elite female tennis serve. J Sports Sci. (2015) 33(7):675–86. 10.1080/02640414.2014.962569 25358037

[82] García-Gonzalez L Moreno MP Moreno A Iglesias D. Relation between knowledge and decision making in tennis players and its influence in sport expertise. Rev Int Cienc Deporte. (2009) 17(5):60–75.

[83] Caserta RJ Young J Janella Cristopher M. Old dogs, new tricks: training the perceptual skills of senior tennis players. J Sport Exerc Psychol. (2007) 229:479–97. 10.1123/jsep.29.4.479 17968049

[84] García L Moreno MP Moreno A Iglesias D Del Villar F. Analysis of the differences in the knowledge of the tennis players, in function del level of expertise. Motricidad Eur J Hum Mov. (2008) 21:31–52.

[85] Vickers JN. Perception, Cognition, and Decision Training. The Quiet Eye in Action. Champaign, IL: Human Kinetics (2007).

[86] Vernon G Farrow D Reid M. Returning serve in tennis: a qualitative examination of the interaction of anticipatory information sources used by professional tennis players. Front Physiol. (2018) 9:895. 10.3389/fpsyg.2018.00895 29951013 PMC6008562

[87] Müller S Abernethy B. Expert anticipatory skill in striking sports: a review and a model. Res Q Exerc Sport. (2012) 83(2):175–87. 10.1080/02701367.2012.10599848 22808703

[88] Timmerman E De Water J Kachel K Reid M Farrow D Savelsbergh G. The effect of equipment scaling on children’s sport performance: the case for tennis. J Sports Sci. (2015) 33(10):1093–100. 10.1080/02640414.2014.986498 25533551

[89] Schmidhofer S Leser R Ebert M. A comparison between the structure in elite tennis and kids tennis on scaled courts (tennis 10s). Int J Perform Anal Sport. (2017) 14(3):829–40. 10.1080/24748668.2014.11868761

[90] Penalva F Guzmán JF Martínez-Gallego R Crespo M. Design and validation of a tennis tool to control on-court technical and tactical training content. Int J Sports Sci Coach. (2021) 17(2):309–17. 10.1177/17479541211027428

[91] Wu J Liu D Guo Z Xu Q Wu Y. Tacticflow: visual analytics of ever-changing tactics in racket sports. IEEE Trans Vis Comput Graph. (2022) 28(1):835–45. 10.1109/TVCG.2021.3114832 34587062

